# Antimicrobial Host Defensins – Specific Antibiotic Activities and Innate Defense Modulation

**DOI:** 10.3389/fimmu.2012.00249

**Published:** 2012-08-14

**Authors:** Hannah Ulm, Miriam Wilmes, Yechiel Shai, Hans-Georg Sahl

**Affiliations:** ^1^Pharmaceutical Microbiology Section, Institute of Medical Microbiology, Immunology and Parasitology, University of BonnBonn, Germany; ^2^Department of Biological Chemistry, The Weizmann Institute of ScienceRehovot, Israel

Current treatment of bacterial and fungal infections heavily relies on strategies which aim to inhibit and kill pathogens with high specificity. These strategies are very successful and antibiotics have contributed to increasing human life expectancy more than any other class of therapeutic drugs. However, antibiotics are losing efficacy as a result of high selection pressure and rapid resistance development. Thus, strategies that rely on boosting natural host defenses are gaining more attention, since compounds targeting host mechanisms should control infections regardless of the antibiotic resistance levels of pathogens. Antimicrobial peptides (AMPs) are considered as ideal candidates for such novel anti-infective strategies since they combine direct antibiotic activities with modulation of immune responses (Figure 1). However, AMPs frequently lack specific molecular targets and tend to have membrane disruptive activities, bearing risks of cytotoxicity. For anti-infective drug development, AMPs should ideally inhibit specific microbial targets without impacting on membranes; peptides with such properties were recently identified in a large subfamily of AMPs, the defensins.

**Figure 1:**
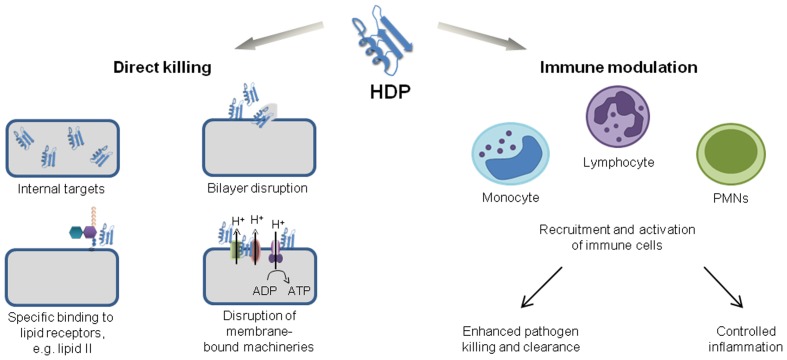
**Functions of HDPs**.

All multicellular organisms produce AMPs to protect surfaces and tissues from invading pathogens. These peptides have been referred to as AMPs and more recently as host defense peptides (HDPs). HDPs are ancient effector molecules of innate immunity with multiple functions. They do not share specific sequence similarities, but can be generally defined as amphiphatic cationic peptides consisting of 12–50 amino acids. They are either linear (e.g., LL-37, magainin, and indolicidin) or have tertiary structures stabilized by disulfide bonds (Hancock and Lehrer, 1998; Shai, 2002; Zasloff, 2002). Defensins *sensu stricto* belong to the latter class and were first isolated from mammals, and subsequently also found in invertebrates and plants.

Plant, fungal, and invertebrate defensins share a common structural motif, the cysteine-stabilized αβ-motif that is composed of an α-helix linked to an antiparallel β-sheet with three or four disulfide bonds; they display either antifungal or antibacterial activity. Recently, it has been demonstrated that antibacterial defensins of fungi and invertebrates bind with high affinity to the bacterial cell wall precursor lipid II. They form an equimolar stoichiometric complex with lipid II, thereby inhibiting the incorporation of the cell wall building-block into the nascent peptidoglycan network (Schmitt et al., 2010; Schneider et al., 2010). NMR-based modeling of the plectasin-lipid II complex indicated that the fungal defensin interacts with the pyrophosphate moiety of lipid II by forming four hydrogen bonds (involving residues F2, G3, C4, and C37). Additionally, a salt bridge between the N-terminus (His18) and the D-glutamic acid in position 2 of the lipid II stem peptide is important for binding (Schneider et al., 2010). Interestingly, the amino acid residues involved in the lipid II binding of plectasin are also present in many other fungal and invertebrate defensins, suggesting a conserved lipid II binding motif.

Cell wall biosynthesis is a prominent target of clinically used antibiotics. For example, the glycopeptide vancomycin, a last-resort antibiotic for treatment of infections with multi-resistant Gram-positive bacteria, binds to the *D*-alanyl-*D*-alanine terminus of the lipid II pentapeptide. However, cross-resistance between vancomycin and plectasin could not be observed and also the presence of *D*-alanine-*D*-lactate found in vancomycin-resistant bacteria did not affect the activity of plectasin (Schneider et al., 2010). In general, only modest resistance development toward HDPs has been observed under *in vitro* selection pressure (Zhang et al., 2005). The lipid II isoprenoid anchor (C_55_P) is also involved in the biosynthesis of other major cell envelope polymers (e.g., wall teichoic acid, capsules). Synthesis of C_55_P-anchored molecules always starts with the transfer of a sugar moiety to the lipid carrier, forming a pyrophosphate linkage. This structural motif is highly conserved, as it is part of several essential building blocks and therefore cannot be easily modified to confer resistance.

The antifungal action of plant and invertebrate defensins also appears to be highly specific and is based on interaction with particular sphingolipids in membranes and cell walls of susceptible fungi. For example, the interaction of RsAFP2 (from radish seeds) with fungal glucosylceramides causes the production of radical oxygen species and apoptosis as well as cell wall stress, septin delocalization, and ceramide accumulation (Thevissen et al., 2012). Other plant defensins such as DmaMp1 (from *Dahlia merckii*) bind specifically to inositol phosphoryl-containing sphingolipids leading to membrane permeabilization and ion efflux (Thevissen et al., 1996, 2003).

In contrast, the activity of vertebrate defensins may be of intermediate specificity for microbial targets with a broader activity spectrum. Vertebrate defensins comprise three subfamilies, α-, β-, and θ-defensins, which differ in their pairing of the six conserved cysteine residues. They are composed of three antiparallel β-sheets and exhibit a broad-spectrum activity against Gram-positive and Gram-negative bacteria, fungi, and some enveloped viruses. α-Defensins have been isolated from the granules of neutrophils and small intestinal Paneth cells whereas β-defensins are mainly expressed in epithelial tissues. The cyclized θ-defensins are found exclusively in leukocytes and bone marrow of Old World monkeys and arose from a pre-existing α-defensin (Ganz, 2003; Schneider et al., 2005). Lipid II binding has also been reported for the vertebrate α-defensin human neutrophil peptide 1 (HNP1) and human-beta defensin 3 (hBD3). However, the affinity of HNP1 to the cell wall precursor is significantly lower compared to that of the fungal peptide plectasin (plectasin-lipid II: 1.8 × 10^−7^ M; HNP1-lipid II: 2.19 × 10^−6^ M; de Leeuw et al., 2010; Sass et al., 2010; Schneider et al., 2010). Besides lipid II sequestration, hBD3 additionally seems to have more generalized effects on membrane bound processes such as electron transport (Sass et al., 2008). These findings indicate that the specificity of lipid II binding correlates to some extent with the antimicrobial spectrum. Defensins with high affinity for lipid II may have evolved to mainly act against Gram-positive bacteria, whereas defensins with lower lipid II affinity may have retained the capacity to interact with additional targets and therefore have a broader antimicrobial spectrum, including Gram-negative bacteria or fungi.

The combination of highly targeted antimicrobial activity with the capacity to positively modulate the immune response is highly attractive as anti-infective strategy. Mammalian HDPs are expressed either constitutively or are inducible in various tissues and cell types, including immune cells like neutrophils or macrophages, as well as keratinocytes and epithelial cells. The expression of these peptides is triggered by conserved microbial structures [lipopolysaccharide (LPS), lipoteichoic acid, CpG oligonuclecotides; via Toll-like receptors (TLRs)] or inflammatory effectors such as cytokines (TNF-α, IL-1β; Zasloff, 2002; Lehrer, 2004; Brown and Hancock, 2006). HDPs have been demonstrated to provide an important link between innate and adaptive immune response, acting as both pro- and anti-inflammatory mediators. They enhance beneficial immune responses and dampen harmful ones, enabling the host to control infections. HDPs modulate the expression of hundreds of genes in immune cells and epithelia, influencing processes like maturation of immune cells, cross-regulation of cytokines/chemokines, wound healing, and angiogenesis. The α-defensins HNP1-3 which are released by tissue invading granulocytes, have been shown to trigger secretion of TNF-α and IFN-γ from macrophages. The cytokine release stimulates the phagocytotic macrophage activity via an autocrine loop, thereby enhancing clearance of opsonized bacteria, as observed *in vitro* and in an murine model (Soehnlein et al., 2008). The β-defensin hBD3 activates professional antigen presenting cells (monocytes, dendritic cells) via TLRs 1 and 2 and thereby stimulates adaptive immune responses (Funderburg et al., 2007). Various defensins recruit immune cells by direct binding to chemokine receptors (CCRs). α-Defensins, for example, enhance the migration of T-cells, while β-defensins exhibit chemoattractant functions for immature dendritic cells, monocytes/macrophages, and mast cells (Yang et al., 2000; Niyonsaba et al., 2002; McDermott, 2004). Furthermore, defensins dampen endotoxin-induced secretion of proinflammatory cytokines by neutralization of extracellular LPS as well as modulation of intracellular signaling pathways (Scott et al., 2002; Mookherjee et al., 2006). Defensins aid in wound healing not only by direct killing of pathogens and boosting of host defense mechanisms, but moreover through stimulation of processes involved in tissue organization. HBD1-4 have been shown to enhance humane keratinocyte migration and proliferation through epidermal growth factor receptor signaling (Niyonsaba et al., 2007). Gene transfer and exogenous expression of hBD3 accelerated closure of infected diabetic wounds in a porcine model (Hirsch et al., 2009), suggesting a therapeutic potential for defensins in wound healing.

Bacterial peptides sharing the overall features of HDPs, i.e., cationic amphiphilicity, such as gramicidin S and polymyxin B have been used in clinics as topical agents. In contrast, no AMP of eukaryotic origin has so far been approved for the treatment of patients. In clinical phase III studies, the HDP-derivatives pexiganan (from *Xenopus laevis* magainin) and iseganan (from porcine protegrin-1) have been shown effective in the prevention of diabetic food ulcer and irradiation-induced oral mucositis, respectively (Trotti et al., 2004; Lipsky et al., 2008). Nevertheless, these substances were not approved for medical use by the US Food and Drug administration. Various other synthetic HDPs are in clinical phase I or II trials, which do not only aim at exploiting the direct antimicrobial features of these peptides, but also their ability to modulate the human immune system (Yeung et al., 2011).

The cationic amphiphilic and peptidic nature of AMPs is often considered unfavorable for development of systemic drugs. However, protease lability, contributing to low serum half-life, may be overcome by different approaches, including the use of peptidomimetics, peptides composed of unusual or D-amino acids (instead of natural L-amino acid), and formulation (e.g., in liposomes; Oren et al., 1997; McPhee et al., 2005). Peptides based on defensin templates have not been investigated in clinical studies so far. Defensins are more protease-resistant due to their disulfide-stabilized structure (Wu et al., 2003; Maemoto et al., 2004), and therefore can have a higher serum half-life as other HDPs mentioned above; e.g., plectasin and its improved derivative NZ2114 showed potent activity in animal models, enhanced serum-stability, and extended *in vivo* half-life (Andes et al., 2009). Also, the plectasin example demonstrates that difficulties associated with high yield production of defensins and with correct cysteine-pairing, can be solved. The use of chemically modified prodrugs, could also improve pharmacokinetics and/or lower toxicity, as in the case of the parental antibiotic colistin (methane sulfonate derivative of polymyxin B; Falagas and Kasiakou, 2006).

Antimicrobial mechanisms based on defined target molecules such as lipid II reduce the risk of unspecific membrane disruption and cytotoxic activities, although HDPs clearly have some specificity for microbial membranes; eukaryotic membranes may be less susceptible due to the absence of anionic lipids on the lipid bilayer surface, the lack of a strong membrane potential gradient and the presence of cholesterol (Hancock and Sahl, 2006). However, it cannot be ignored that certain HDPs display potential harmful effects like degranulation of mast cells and enhancement of apoptosis (Niyonsaba et al., 2001; Barlow et al., 2006). It has been reported that hBD3 promotes the proliferation of oral carcinoma and osteosarcoma cells acting as a potential proto-oncogene (Kesting et al., 2009; Kraus et al., 2012). HDPs are reminiscent of peptides with nuclear localization signals and many peptides can migrate into the cell core; the cathelicidin LL-37 was even demonstrated to have nuclear translocation ability regarding DNA plasmids (Sandgren et al., 2004). Thus, it is obvious that such activities need to be extensively studied and taken into account for any drug development program.

The increasing knowledge of the importance of immunomodulatory HDP functions, has led to the synthesis of so called innate defense regulator peptides (IDRs; Easton et al., 2009). These are small synthetic peptides derived from HDP templates, which were designed to selectively modulate the innate immune system without the detrimental activities displayed by certain natural HDPs (see above). Several recent studies focused on cathelicidin-derived IDRs (Choi et al., 2012). The synthetic peptides IDR-1 and IDR-1002 (from bovine bactenecin), despite lacking direct antimicrobial activity, were shown to confer protection against systemic bacterial infection in mouse models challenged with methicillin-resistant *S. aureus* and vancomycin-resistant enterococci. Notably, these IDRs combine anti-infective and anti-inflammatory properties. IDR-1 and IDR-1002 contribute to bacterial clearance by inducing chemokines secretion and enhancing leukocyte recruitment. Moreover, the peptides suppress the induction of several proinflammatory cytokines, thereby dampening immune-mediated inflammation and preventing tissue damage (Scott et al., 2007; Nijnik et al., 2010; Wieczorek et al., 2010; Turner-Brannen et al., 2011). IMX-942, which is based on IDR-1, is tested for its ability to help combat nosocomial infections in immune-suppressed cancer patients, and has recently completed clinical phase I trials^1^. The HLA-I-derived decapeptide RDP58 inhibits the synthesis of proinflammatory cytokines like TNF-α, IL-2, IL-12, and IFN-γ by interfering with MyD88-signaling (Travis et al., 2005). RDP58 has proven safety and efficacy in clinical phase II studies with inflammatory bowel disease patients^2^.

Taken together, it appears a most promising approach to design future anti-infective drugs that target host defenses and may combine this with targeted antibiotic activities, even more since classic antibiotics such as macrolides also appear to have immune modulatory properties (Tauber and Nau, 2008). On the other hand, it is obvious that for systematic exploitation of this concept, we need to know more about both, the molecular mechanisms underlying the immune modulation and about specific, targeted antibiotic activities of HDPs – it would be rather surprising if these would occur only with defensins.

## Footnotes

^1^http://www.inimexpharma.com

^2^http://www.genzyme.com
